# *S-Equol* ameliorates insulin secretion failure through Chrebp/Txnip signaling via modulating PKA/PP2A activities

**DOI:** 10.1186/s12986-020-0426-8

**Published:** 2020-01-14

**Authors:** Ka Chen, Hedong Lang, Li Wang, Kai Liu, Yong Zhou, Mantian Mi

**Affiliations:** 1 Research Center for Nutrition and Food Safety, Institute of Military Preventive Medicine, Third Military Medical University, Chongqing, 400038 People’s Republic of China; 2 Department of Clinic Nutrition, People’s Hospital of Chongqing Banan District, Chongqing, 401320 People’s Republic of China

**Keywords:** *S-Equol*, Diabetes, Insulin secretion, Chrebp, Txnip, PKA, PP2A

## Abstract

**Background:**

*S-Equol*, produced from daidzein by gut microbiota, has been suggested as an potential anti-diabetic agent, but the underlying mechanisms remain unclear. Recent evidences demonstrated that carbohydrate response element-binding protein (Chrebp)/Thioredoxin-interacting protein (Txnip) signaling played central roles on diabetes progression, particularly in relation to the function maintenance and apoptosis of pancreatic β-cell. Here, we investigated the effects of *S-Equol* on β-cell function and Chrebp/Txnip signaling*.*

**Methods:**

Zucker diabetic fatty rats were treated with racemic *Equol* (120 mg/kg.BW.d) for 6 weeks. The glucose and lipid metabolism were monitored during the supplementation, and the Chrebp and Txnip expression were measured by using Western blotting. INS-1 cells were incubated with high glucose (26.2 mM) with or without *S-Equol* (0.1 μM, 1 μM, 10 μM) for 48 h. Glucose-stimulated insulin secretion (GSIS) was evaluated by radioimmunoassay, and the apoptosis of INS-1 cells was analyzed using Annexin V-FITC/PI and TUNEL assay. The dual luciferase reporter assay, chromatin immunoprecipitation assay and Western-blotting followed by Chrebp small interfering RNAs were utilized to clarify the mechanism of transcriptional regulation of *S-Equol* on Chrebp/Txnip signaling and the activities of protein kinase A (PKA) and protein phophatase (PP2A) were also detected.

**Results:**

In vivo, *Equol* supplementation delayed the onset of the hyperglycemia and hyperlipemia, ameliorated insulin secretion failure, enhanced GSIS in isolated islets, and significantly reduced Chrebp and Txnip expression in islets. In vitro, *S-Equol* treatment enhanced GSIS of high glucose cultured INS-1 cell, and reduced apoptosis of INS-1 cells were also observed. Moreover, *S-Equol* dramatically suppressed Txnip transcription, as evident by the reduction of Txnip protein and mRNA levels and decrease in the *Txnip* promoter-driven luciferase activity. Meanwhile, *S-Equol* significantly inhibited Chrebp/Mlx expression and decreased occupancy of Chrebp on the *Txnip* promoter, and combined with si*Chrebp,* we confirmed that *S-Equol* improvement of insulin secretion was partially through the Chrebp/Txnip pathway. Furthermore, *S-Equol* significantly decrease nuclear translocation of Chrebp, which was related with the decrease activity of protein kinase A (PKA) and the increase activity of protein phophatase (PP2A).

**Conclusions:**

*S-Equol* could ameliorate insulin secretion failure, which was dependent on the suppression of Chrebp/Txnip signaling via modulating PKA/PP2A activities.

## Background

As the prevalence of type 2 diabetes mellitus (T2DM) continues to increase worldwide, identifying dietary factors that can help modulate the insulin sensitivity and insulin secretion process is an important public health and clinical goal for reducing T2DM burden (1). Increasing evidences indicate that dietary soy as well as the isoflavone daidzein or *S-Equol* could improve glycemic control and insulin responsiveness (2, 3), but the conclusions are still uncertain. *S-Equol*, the end product of daidzein by intestinal bacteria, attracts tremendous attention as its superiority to other isoflavones in its estrogenic and antioxidant activity (4–6). Moreover, results of recent studies (2, 7–10) suggest that *S-Equol* may affect glucose and lipid metabolism in ways that were independent of its estrogenic activity, for instance, via increased energy expenditure, decrease release of adipokines, activation of peroxisome proliferator–activated receptor and AMP-activated protein kinase signaling. Thus, the mechanisms whereby *S-Equol* exerts its beneficial effects should be further elucidated.

Defects in glucose-stimulated insulin secretion (GSIS) by pancreatic β-cells are central to T2DM risk and progression (1, 3, 11). Either increased proliferation or decreased cell death in β-cells directly enhance insulin secretion (1, 3, 11). Thioredoxin-interacting protein (Txnip), a member of the arrestin family, involves various cellular processes including redox state, inflammation and apoptosis (12–14). Notably, recent studies have also revealed that Txnip is a potent inhibitor of cellular glucose uptake and aerobic glycolysis (15), and plays a particularly key role in hyperglycemia-induced β cell apoptosis and diabetes development (13, 14, 16–18). High glucose and diabetes induce Txnip expression, whereas inhibition of Txnip expression or Txnip deficiency protects against pancreatic β cell apoptosis and diabetes (11, 19). Glucose activates Txnip transcription by recruiting the carbohydrate response element-binding protein (Chrebp) and its obligate transcription partner Mlx to the *Txnip* promoter (18, 20, 21). Moreover, recent studies have discovered that the activity of Chrebp is regulated by its phosphorylation status and cellular localization (20, 22–24). High glucose stimulates *Chrebp* gene expression and also stimulate its translocation from the cytosol to the nucleus, thereby increasing its DNA-binding/transcriptional activity (25, 26). In light of Txnip in pathological process in T2DM and the central role of Chrebp in the regulation of Txnip, here we investigated whether *S-Equol* affects insulin secretion and influences the Chrebp/Txnip signaling both in vivo and in vitro*,* which would support new evidences to demonstrate the effects and mechanisms of S-Equol on diabetes.

## Methods

### Animals and the experimental procedures

A total of 12 male Zucker diabetic fatty (ZDF) rats (*fa/fa*) and 12 male Zucker lean (ZLR) rats (*fa/+) *were purchased from Vital River Laboratories International Inc. (Beijing, China). Rats were scheduled to arrive at 6 weeks of age, and housed two per cage under a 12-h light/dark cycle at controlled temperature conditions with ad libitum access to Purina 5008 chow and water. All animal experiments were approved by the Third Military Medical University Committee for animal research and followed our institutional guidelines for the use of laboratory animals.

Following 2 weeks of adaptation, the rats at 8 weeks of age were randomly assigned to four groups: ZLR rats with no drug treatment (ZLR; *n* = 6), ZLR rats with *Equol* supplementation (Eq + ZLR; n = 6), ZDF rats with no drug treatment (ZDF; n = 6), ZDF rats with *Equol* supplementation (Eq + ZDF; n = 6). *Equol* (purity > 98%; a racemic mixture of *Equol*) was purchased from Nanjing Zelang Biotechnology Co. Ltd., China, and *Equol* were dissolved in distilled water and administered intra-gastrically (120 mg/kg.bw) at 9:00 am every day. Body weight, food and water intake were measured every 2 days, blood glucose once weekly, and oral glucose tolerance test (OGTT), serum levels of insulin every 2 weeks. After 6 weeks of treatment, all the rats were sacrificed and total triglyceride (TG), total cholesterol (Tch), low density lipoprotein (LDL), high density lipoprotein (HDL), C-peptide, Glucagon, and *S-Equol* levels were detected. Meanwhile, pancreatic islets (3 in each group) were isolated and cultured overnight for insulin secretion studies as previous described (11); the other pancreatic islets were harvested and stored at − 80 °C to use in the following experiments of this study.

### OGTT

Rats were fasted for 16 h prior to OGTT to allow complete drug washout. An oral glucose load of 2 g/kg body weight was administered. Blood samples were collected from the tail vein at 0, 15, 30, 60, and 120 min after glucose loading. Blood glucose levels were measured using an Accu-check glucose analyzer (Roche, Germany) and overall changes in glucose during OGTT were calculated as areas under the curve (AUC). Meanwhile, using a radioimmunoassay, the blood insulin levels during OGTT at each time point were measured (Linco Research, MO, USA).

### Serum indicators measurements

Serum Tch, TG, HDL, LDL levels were measured by an automatic biochemistry analyzer (Olympus AU5400, Tokyo, Japan). Commercial assay kits were used to determine plasma Glucagon (Rat Glucagon ELISA kit, WAKO, Japan), C-peptide (Rat C-peptide ELISA; ALPCO) levels were measured using ELISA kits. *S-Equol* was measured by using HPLC method as the previous study (8).

### Glucose-stimulated insulin secretion (GSIS) from isolated islets

Duplicate samples of three isolated islets were incubated to measure insulin secretion in Dulbecco’s modified Eagle’s medium (Gibco, USA) with low (2.8 mM) or high (16.7 mM) glucose concentrations for 2 h at 37 °C. Supernatants were collected and insulin remaining in the islets was extracted with 3% acetic acid, then insulin was measured using a radioimmunoassay (Linco Research, MO, USA). The total islet insulin content included both the insulin secreted into supernatant and the remaining in the islet. Amplification rate of insulin secretion was calculated as the ratio of insulin release level at high glucose compared with basal glucose stimulation.

### Cell culture and the experimental procedures

Rat insulinoma beta cells (INS-1) were maintained in RPMI 1640 medium (Gibco-Invitrogen, Carlsbad, CA, USA) containing 10% fetal bovine serum (HyClone, USA), 1% penicillin/streptomycin, 1 mM sodium pyruvate, 2 mM L-glutamine, 10 mM HEPES, and 0.05 mM 2-mercaptoethanol in a 37 °C incubator at 5% CO_2_. All experiments were performed on the cells when the cells reached 75–85% confluence. To detect the effect of *S-Equol* on high glucose-treated INS-1 cells, cells were incubated in RPMI medium containing 26.2 mM glucose with or without *S-Equol* [CAS No: 531–95-3, Dasailu pharmaceutical chiral technology (Shanghai) Co., LTD] at the indicated concentrations (0.1 μM, 1 μM, 10 μM) for 48 h.

### Glucose-stimulated insulin secretion in INS-1 cells

INS-1 cells were seeded (5 × 10^5^ cells/well) in 12-well plates. After 48-h treatments, INS-1 cells were washed and incubated in KRB-HEPES with 0.1% BSA for a quiescent period of 90 min. Then, the cells were incubated in KRB-HEPES-BSA containing 2.8 or 16.5 mM glucose for 90 min to respectively measure the basic insulin secretion (BIS) and GSIS. Insulin secreted into the medium was evaluated by the radioimmunoassay. The total cell insulin content was calculated by adding insulin secreted into the supernatant plus that remaining in the cells, with correction by cell number in each group.

### Quantitative real-time PCR (qRT-PCR)

Total RNA was extracted with Trizol buffer and reverse transcribed into cDNA using the BioRT cDNA first strand synthesis kit (Boaosen, Beijing, China) following the manufacturer’s recommendations. Quantitative real-time PCR was performed with the Bioeasy SYBR Green Real-time PCR Kit (Boaosen, Beijing, China) on the MyiQ Real-time PCR system (Bio-Rad, USA). All data were analyzed using the mRNA expression of β-actin as an internal reference. The primer sequences used were as follows: P*roinsulin* (*PPI;* forward 5′- GTGTGGGGAACGTGGTTTCT-3′; reverse 5′-TGCCAAGGTCTGAAGATCCC -3′); *Txnip* (forward 5′-TGTGTGAAGTTACTCGTGTCAAA-3′; reverse 5′- GCAGGTACTCCGAAGTCTGT-3′); *β-actin* (forward 5′-ACCAACTGGGACGATATGGAGAAGA-3′; reverse 5′-ACGACCAGAGGCATACAGGGACAA-3′).

### Western blotting analysis

Total proteins were extracted with T-PER Protein Extraction kit (Pierce Biotechnology, Inc., Rockford, IL, USA), and nuclear fractions were collected using the Nuclear Protein Extraction Kit (Sigma-Aldrich, MO, USA) according to the manufacturer’s instructions. Protein concentrations were determined using the BCA Protein Assay Kit (Beyotime, China). Protein samples were separated by 12% SDS-PAGE and transferred to PVDF membranes (Millipore, Billerica, MA, USA). After blocking with 5% BSA at room temperature for 1 h, the membranes were incubated overnight at 4 °C with the following primary antibodies: Glucose transporter 2 (Glut2; Santa Cruz Biotechnology, CA, USA),Uncoupling protein-2 (UCP-2; Abcam, Cambridge, UK), Cherbp (Novus, Oakville, Canada) and Txnip (Novus, Oakville, Canada). β-Actin (Beyotime, Beijing, China) was used as a loading control for total proteins and Lamin A (Cell Signaling Technology, MA, USA) for nuclear proteins. Then membranes were washed and incubated in appropriate secondary antibody for 1 h. The target proteins were detected using chemiluminescence system (Pierce Biotechnology), and the immunoblots were quantified by densitometry using Bio-Rad Quantity One 4.4.0 software (Bio-Rad Lab., Hercules, CA, USA).

### Annexin V-FITC/PI assay

Apoptosis was detected using an Annexin V-FITC/PI detection kit (Bipec Biopharma Corp., USA) according to the manufacturer’s instructions. Briefly, cells were harvested, washed and resuspended in binding the buffer. Then cells were mixed with AnnexinV-FITC and propidium iodide (PI) for 15 min at room temperature in the dark. Fluorescence was detected by flow cytometry (FACS-400, USA). The percentage of apoptosis was reflected by the number of Annexin V (+)/PI (−) cells relative to the number of Annexin V (+)/PI (+) cells.

### Terminal deoxynucleotidyl transferase dUTP nick end labeling (TUNEL) assay

Apoptotic INS-1 cells were also studied using TUNEL assay. INS-1 cells were seeded on coverslips. After treatment, cells were washed with PBS, fixed in 4% paraformaldehyde, permeabiliz ed. with 0.1% Triton X-100 and blocking with 5% BSA. Fluorescein in Situ Cell Death Detection kit (Millipore cat. #S7110, Billerica, MA, United States) was used according to the manufacturer’s protocol. Six different fields in each coverslip and six samples per group were used to collect the images and the quantification of TUNEL positive cells and Apoptotic rate. The images were obtained using a confocal microscope (Zeiss LSM 510 META; Carl Zeiss, Oberkochen, Germany).

### *Txnip* promoter activity assay

INS-1 cells were plated in 96-well plates and incubated until the cells reached 80% confluence. The rat *Txnip* promoter-luciferase reporter (− 500 to 279 bp) plasmid constructs were constructed as the previous study (27). INS-1 cells were transfected with the *Txnip* promoter-luciferase reporter plasmid or the empty vector (internal control) using Lipofectamine 2000 (Invitrogen, Carlsbad, CA, USA) following the manufacturer’s protocol. After transfection, cells were incubated in high glucose conditions with or without *S-Equo*l for 48 h. Luciferase activity was analyzed using the Dual-Luciferase Assay Detection kit (Promega, Madison, WI, USA) with a luminometer microplate reader (Molecular Devices, USA) according to the manufacturer’s instructions. Normalized luciferase values were expressed as the percentage relative to the control group.

### Chromatin immunoprecipitation (ChIP) assay

ChIP assay was performed using a ChIP assay kit (Cell Signaling Technology) following the manufacturer’s instructions. After the indicated treatment, cells were harvested. After crosslinking the chromatin in 1% formaldehyde at 37 °C for 10 min and neutralizing with glycine for 5 min at room temperature, the cells were washed with cold PBS and lysed in sodium dodecyl sulfate lysis buffer. Subsequently, the chromatin was sonicated to generate an average length of 150–900 bp DNA fragments. Immunoprecipitation of crosslinked protein/DNA was performed overnight at 4 °C with the following antibodies: Chrebp (Santa Cruz Biotechnology) and normal rabbit IgG (as a negative control; Cell Signaling Technology). The purified DNA fragments were quantified by PCR using primers 5′-CGCACCCGAACAACAACCAT-3′ (forward) and 5′- AAGCGGGAGCCGGAAACGG-3′ (reverse) to determine the binding of Cherbp to *Txnip* promoter.

### *Chrebp* small interfering RNA and transfection

Predesigned *Chrebp* siRNA and control scramble siRNA (as the negative control) were synthesized by Invitrogen. The stealth RNAi molecules (40 nM) were transfected into INS-1 cells using Lipofectamine 2000 according to the manufacturer’s instructions. The ability of the stealth RNAi oligonucleotide to knockdown Chrebp expression was analyzed by Western- blotting using the total cell extract.

### Detection of the activities of protein kinase a (PKA) and protein phosphatase 2A (PP2A)

INS-1 cells were collected and cell lysate was used to detect PKA activity using a PKA Kinase Activity Assay Kit (Abcam, Cambridge, UK) according to the manufacturer’s instructions. PP2A activity was measured using a Serine/Threonine Phosphatase Assay Kit (Promega, Madison, USA) following the manufacturer’s instructions.

### Statistics

All experiments were performed at least three times. Data were presented as the mean ± standard deviation (SD). Different groups were compared by one-way analysis of variance (ANOVA) followed by Tukey-Kramer post hoc tests for multiple comparisons. A value of *P* < 0.05 was considered statistical significant.

## Results

### *S-Equol* improves hyperglycemia and hyperlipemia in ZDF rats

**Fig. 1:**
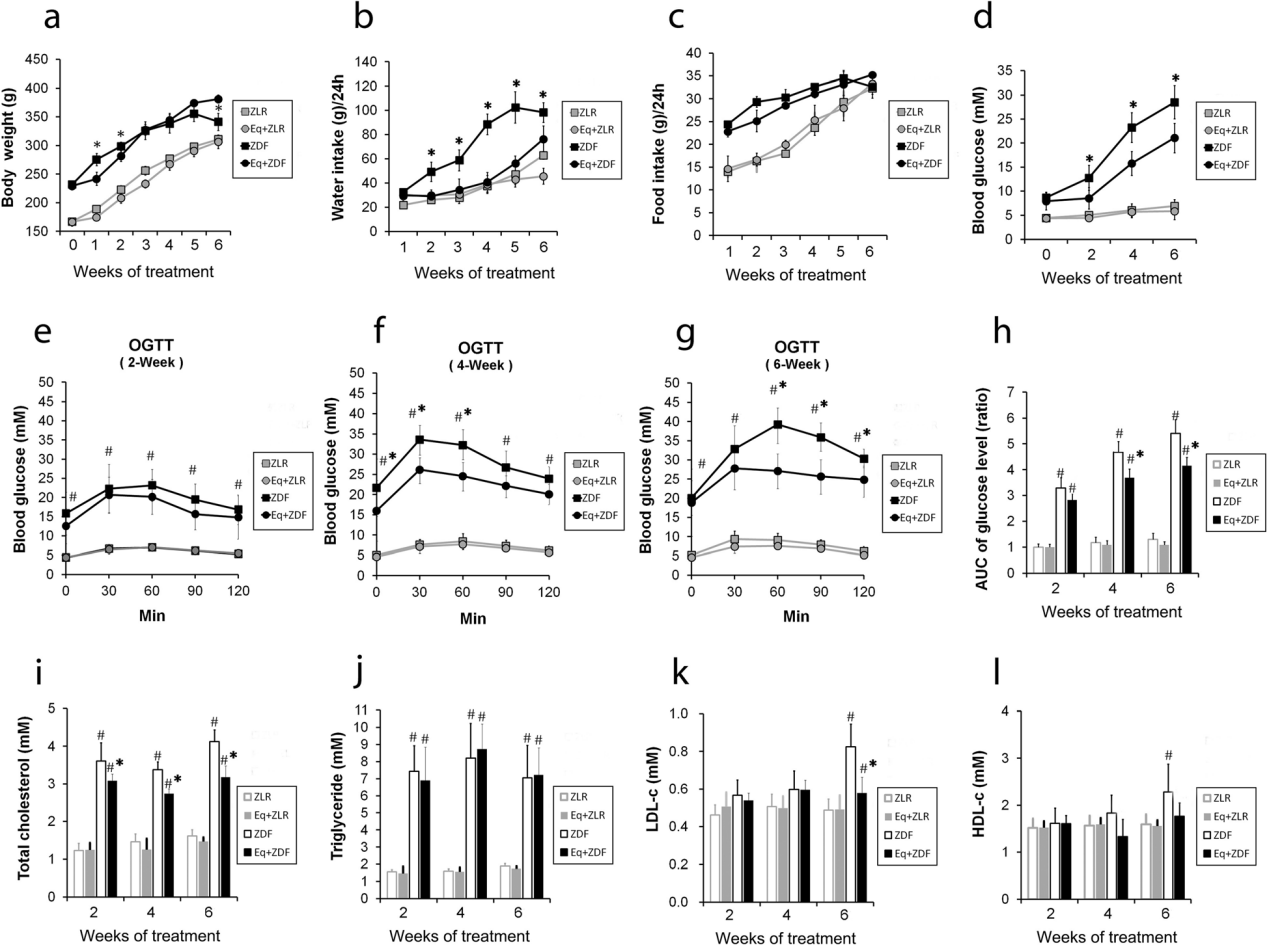
*S-Equol* improves hyperglycemia and hyperlipemia in ZDF rats. The ZDF rats were treated with or without *Equol* (120 mg/kg.BW.d) for six weeks, and ZLR rats were used as the control. Body weight (**a**), water intake (**b**), food intake (**c**), fasting blood glucose (**d**), OGTT test after 2-week (**e**), 4-week (**f**) and 6-week (**g**) treatment and the AUC of glucose level (**h**), total cholesterol (**i**), triglyceride (**j**), LDL-c(**k**) and HDL-c (**I**) were assessed. Eq, *Equol*; ZDF, Zucker diabetic fatty rats (*fa/fa*); ZLR, Zucker lean rats (*fa/+*); OGTT, oral glucose tolerance test; AUC, areas under the curve; LDL, low density lipoprotein; HDL, high density lipoprotein. Data are presented as mean ± S. D (*n* = 6), ^#^ *P* < 0.05 *vs.* the ZLR group; * *P* < 0.05 *vs.* the ZDF group

To determine how *S-Equol* involved in the development of diabetic phenotypes in ZDF rats, we observed the effects of *Equol* (120 mg/kg.bw.d) treatment on body weight, water intake, food intake, serum glucose level, and serum lipid profile. During the first 3 weeks of the supplementation, relatively lower body weights were seen in ZDF rats with *Equol*, when compared to ZDF rats without *Equol* feeding (Fig. 1a). However, the difference in body weight were disappeared in the following 3 weeks, and after 6 weeks treatment, the body weights of ZDF rats fed with *Equol* kept increasing while ZDF rats without *Equol* turns to lose weight (Fig. 1a). Meanwhile, the amount of water intake in ZDF rats with *Equol* was less than that of ZDF rats without *Equol* in the whole supplementation period (Fig. 1b), but there were no differences in the amount of food intake (Fig. 1c). Serum glucose levels of ZDF rats given *Equol* were significantly reduced compared to the ZDF rats in the whole supplementation period of this study (Fig. 1d). Furthermore, OGTT revealed significant amelioration of glucose metabolism in ZDF rats with *Equol* treatment (Fig. 1e-h). The changes in Tch, TG, LDL-c, HDL-c were shown in Fig. 1i-l, which indicated that *Equol* supplementation led to a significant reduction of Tch and LDL in ZDF rats. These results suggest that *Equol* supplementation improves glucose and lipid metabolism and enhances glucose tolerance in ZDF rats.

Additionally, as *S-Equol* was the bioactive form of *Equol*, we detected the plasma level of *S-Equol* by using HPLC assay. After the 6 weeks’ supplementation, plasma levels of *S-Equol* in *Equol* supplemented ZDF rats reached at 7.04 ± 1.27 μM and 5.31 ± 0.86 μM in ZLR rats, whereas only 0.53 ± 0.24 μM and 0.47 ± 0.31 μM in the ZLR rats and ZDF rats without *Equol* supplementation.

### *S-Equol* supplementation improves insulin secretion in ZDF rats

**Fig. 2:**
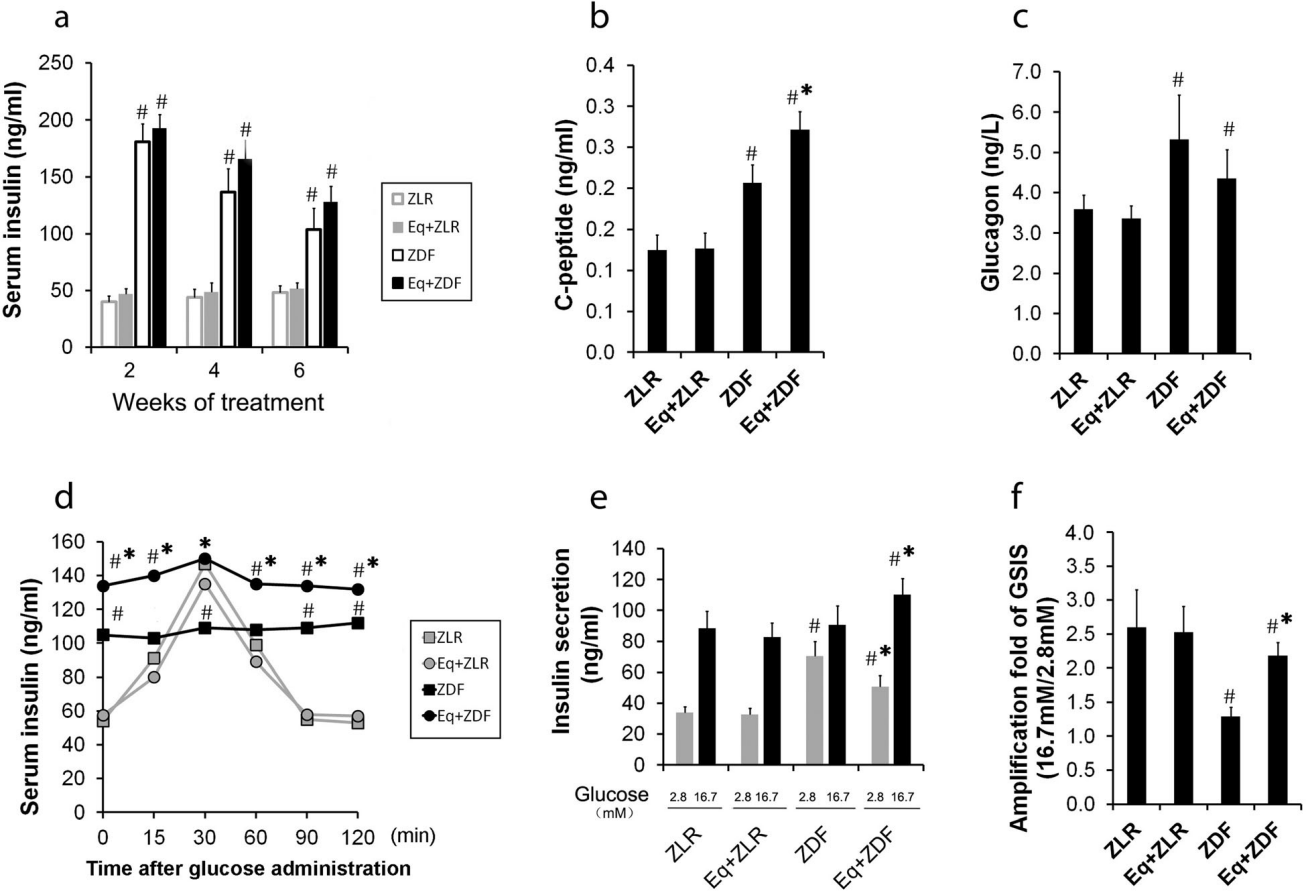
*S-Equol* ameliorates insulin secretion failure in ZDF rats*.* ZDF rats were treated with or without *Equol* (120 mg/kg.BW.d) for six weeks, and ZLR rats were used as the control. (**a**) Serum insulin concentrations after 2, 4 and 6 weeks treatment; (**b**) Serum C-peptide and (**c**) glucagon levels after 6 weeks treatment; (**d**) Serum insulin levels during OGTT after 6 weeks treatment. Following an overnight fast, rats were injected with glucose (2.0 g/kg.BW). Insulin values were assessed at 0, 15, 30, 60, 90 and 120 min during OGTT. (**g**) GSIS from isolated islets. Batches of 3 isolated pancreatic islets were stimulated with 2.8 mm or 16.7 mm glucose for 30 min. Amplification rate of insulin secretion (**h**) at high (16.7 mM) glucose compared with basal (2.8 mM) glucose stimulation for 30 min. Eq, *Equol*; ZDF, Zucker diabetic fatty rats (fa/fa); ZLR, Zucker lean rats (fa/+); GSIS, glucose stimulated insulin secretion. Data are presented as means ± S. D (n = 6), ^#^ *P* < 0.05 *vs.* the ZLR group; * *P* < 0.05 *vs.* the ZDF group

ZDF rats showed enhanced insulin secretion after 2 weeks treatment, and with the progression of obesity, insulin secretion decreased after 6 weeks treatment (Fig. 2a). Compared with ZDF rats, marginally significant higher levels of serum insulin were observed in ZDF rats supplemented with *Equol* after 2, 4 and 6 weeks’ treatment (Fig. 2a) and significant higher levels of C-peptide were detected in *Equol* supplemented ZDF rats after 6 weeks treatment (Fig. 2b)*.* Meanwhile, slightly reduction of serum glucagon were detected in *Equol* supplemented ZDF rats after 6 weeks treatment (Fig. 2c). Furthermore, during OGTT tests, serum insulin levels at each time point were higher in *Equol*-treated-ZDF rats compared with those without *Equol* (Fig. 2d). To evaluate the effect of *Equol* on insulin secretion ex vivo, isolated pancreatic islets were used to determine GSIS. As shown in Fig. 2 e-f, insulin secretion from ZDF rat islets in response to high glucose was blunted, but was improved by *Equol*. These results suggest that *Equol* ameliorates insulin secretion failure in ZDF rats both in vivo and ex vivo.

### *S-Equol* treatment alleviates GSIS in high-glucose cultured INS-1 cells

**Fig. 3:**
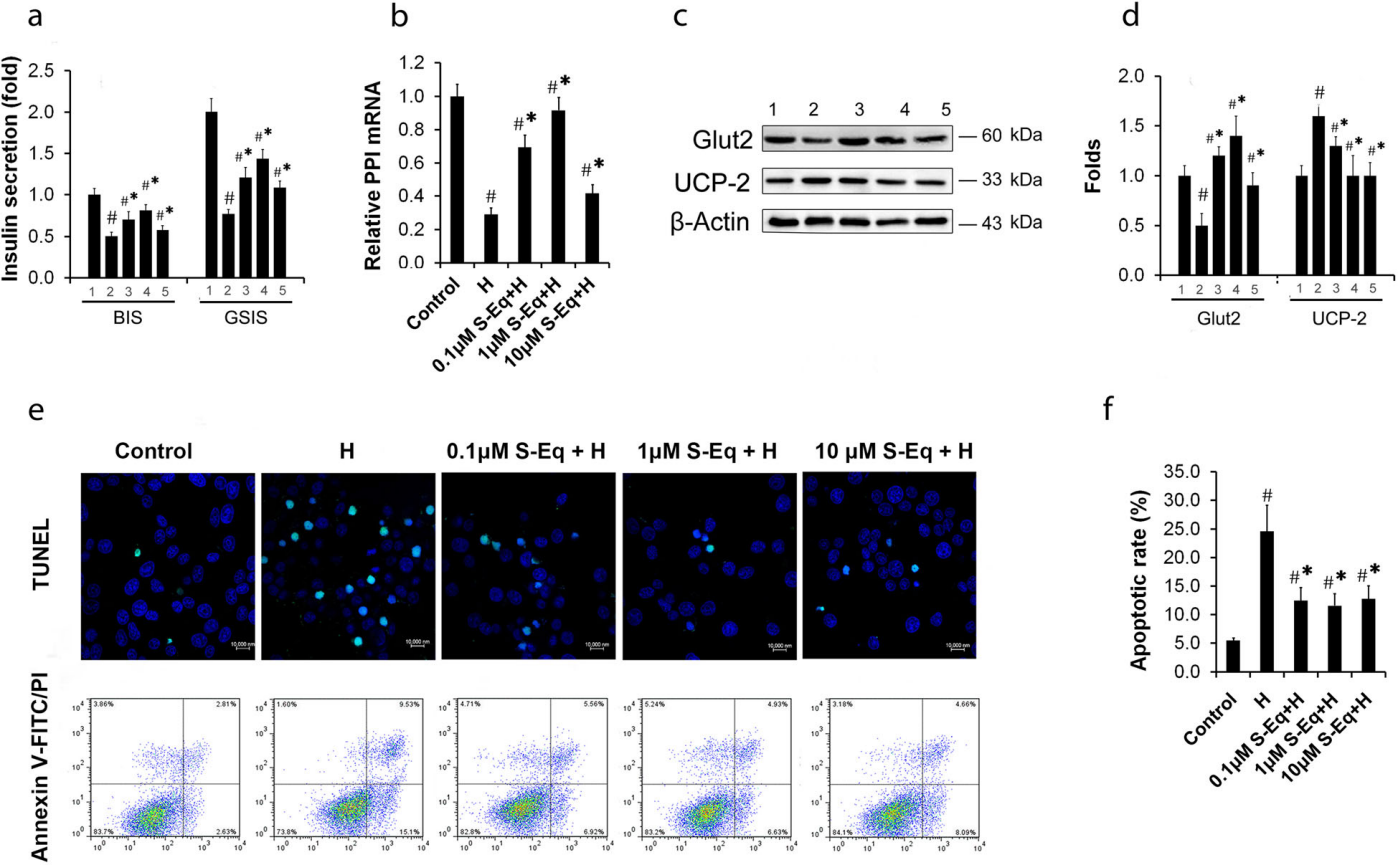
*S-Equol* enhances insulin secretion and inhibits apoptosis from INS-1 cells cultured with high glucose. INS-1 cells were treated for 48 h with or without *S-Equol* (0.1, 1 and 10 μM) in the continuous presence of high glucose (26.2 mM), and cells cultured with 5 mM of glucose denote the control. (a) GSIS assay. After the treatment of INS-1 cells for 48 h, the basic insulin secretion (BIS) and GSIS were detected respectively in INS-1 cells exposed to 5 or 25 mM glucose medium for 90 min in the continuous presence or absence of *S-Equol* after a quiescent period of 90 min; (b) *PPI* mRNA detection by real time-PCR; (c) The expression of Glut2 and UCP-2 was measured by Western blotting analysis and quantified based on the changed folds normalized to their control groups; (d-e) Apoptosis cell assay of INS-1 cells by TUNEL method, Annexin V method and apoptotic rate calculation in INS-1 cells. Lane “1 – 5” represents Control, H, 0.1 μM *S-Eq* + H, 1 μM *S-Eq* + H and 10 μM *S-Eq* + H group respectively. *S-Eq, S-Equol;* H, High glucose; GSIS, Glucose stimulated insulin secretion; BIS, basic insulin secretion. *PPI, Preproinsulin*; Glut2, Glucose transporter 2; UCP-2, Uncoupling protein-2. Five to eight independent experiments were run in each experiments and results were presented as means ± SD. *P < 0.05 *vs. *the Control group, ^#^ *P* < 0.05 *vs.* the H group

To further identify the effects of *S-Equol* on insulin secretion, serial experiments with INS-1 cells cultured in high glucose were done. Insulin secretion in response to 5 or 25 mM of glucose was measured from INS-1 cells (Fig. 3a). As previously reported (3), insulin release in response to 5 or 25 mM of glucose was drastically reduced for cells cultured at high glucose as compared with the control cells. When *S-Equol* (0.1, 1 or 10 μM) was included during the culture of INS-1 cells with high glucose, insulin secretion in response to 5 or 25 mM glucose significantly increased as compared with the cells cultured in the absence of *S-Equol*. Meanwhile, to further analyze specific effects on insulin production, real-time PCR analysis for *PPI* mRNA (Fig. 3b) and Western blotting analysis for Glut2 and UCP-2 were performed (Fig. 3 c-d). Compared with the high glucose cultured INS-1 cells, INS-1 cells treated with *S-Equol* showed significant increase in their cellular *PPI*-mRNA content and Glut2 protein and decrease in UCP-2 protein (Fig. 3b-d).

### *S-Equol* increases cell proliferation and inhibits apoptosis of INS-1 cells cultured in high glucose

The deterioration of GSIS in β-cells exposed to elevated levels of glucose is connected with the loss of β-cell mass (1, 3). In the present study, we sought to determine whether the observed beneficial effect of *S-Equol* on improved secretion response was related to the increased cell proliferation or the reduced apoptosis of INS-1 cells. As the results of MTT tests, *S-Equol* (0.1, 1, 10 μM) treatment had improved cell viability of INS-1 cells cultured in high glucose with 78.55, 88.93 and 64.78% respectively, compared with 42.04% in INS-1 cells cultured in high glucose in the absence of *S-Equol*. Next, we examined the presence of apoptosis in normal glucose and high glucose-treated cells with or without *S-Equol*. Both TUNEL assay and Annexin-V staining showed significantly increase apoptotic cells by glucotoxicity and decreased by treatment with *S-Equol* (Fig. 3e-f).

### *S-Equol* supplementation suppresses Chrebp/Txnip signaling in vivo and in vitro

**Fig. 4:**
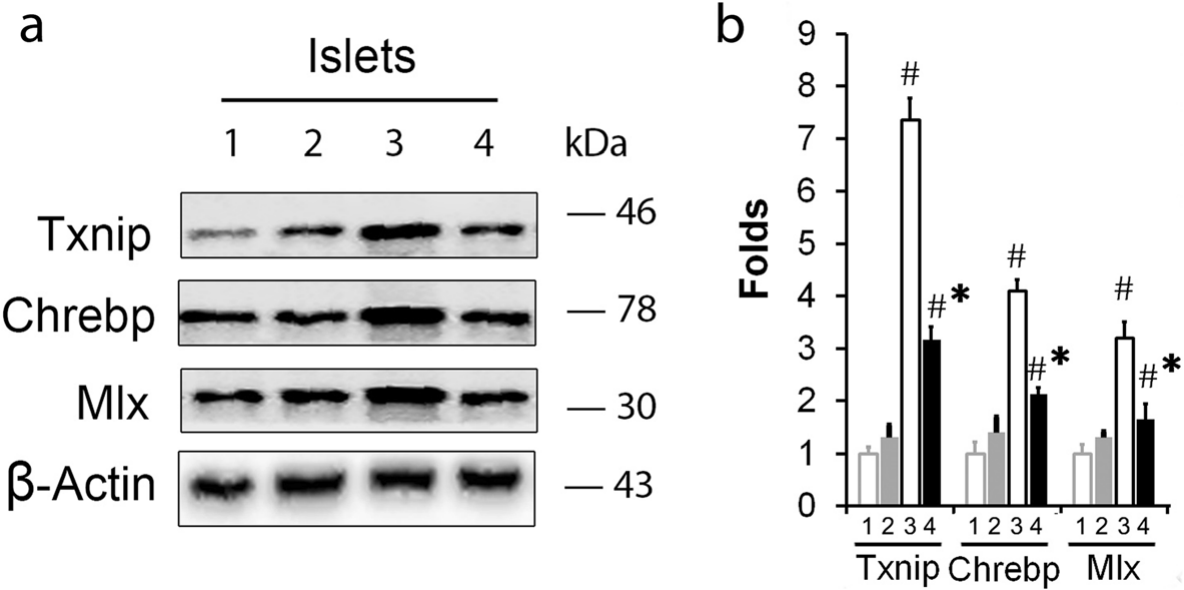
*S-Equol* regulates Chrebp /Txnip signaling in ZDF rats. (**a**) Western blotting to determine Txnip, Chrebp and Mlx expression in ZDF rats with or without *Equol* (120 mg/kg.BW.d). (**b**) Densitometric quantification of the relative folds of the proteins /actin ratio in ZDF rats was shown in bar graph. ZDF, Zucker diabetic fatty rats (*fa/fa*); ZLR, Zucker lean rats (*fa/+). *Lane “1 – 4” represents ZLR, Eq + ZLR, ZDF and Eq + ZDF group respectively. Three independent experiments were run and results were presented as means ± SD. ^#^ *P* < 0.05 *vs.* the Control group, * *P* < 0.05 *vs.* ZDF group

**Fig. 5:**
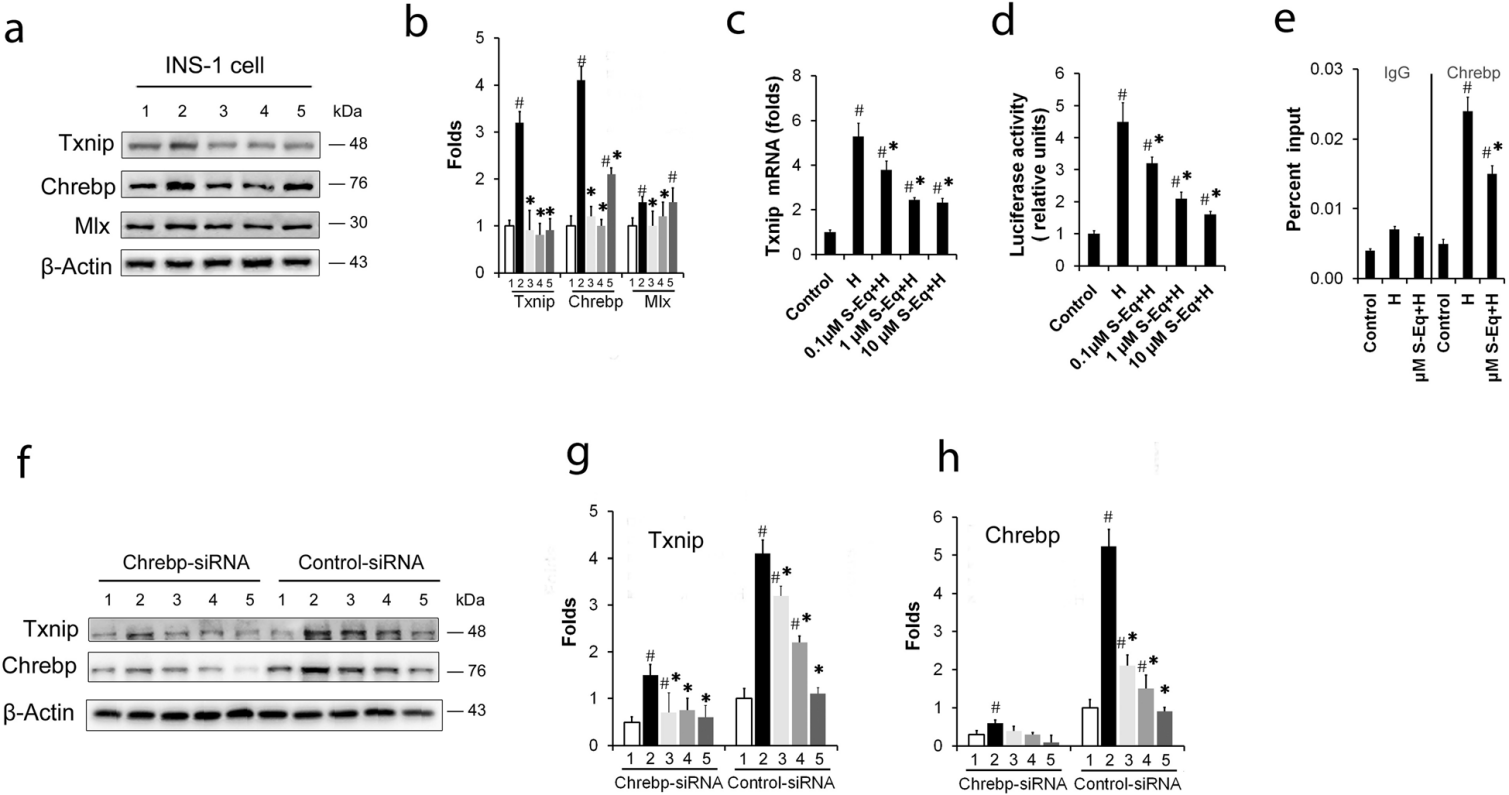
*S-Equol* regulates Chrebp/Txnip signaling in high glucose cultured INS-1 cells. INS-1 cells were treated for 48 h with or without *S-Equol* (0.1, 1 and 10 μM) in the continuous presence of high glucose (26.2 mM), and cells cultured with 5 mM of glucose denote the control. (**a**) Western blotting analysis to determine Txnip, Chrebp and Mlx, and (**b**) densitometric quantification of the relative folds of the proteins /actin ratio in INS-1 cells was shown in bar graph; (**c**) Quantitative RT-PCR analysis of *Txnip*-mRNA transcription; (**d**) *Txnip* promoter activities. Luciferase activity was expressed as the percentage relative to the control group; (**e**) ChIP assay. Cell lysate was precipitated with anti-Chrebp and normal IgG was used as a negative control. (**f** - **h**) The effect of si-*Chrebp* on protein levels of Txnip and Chrebp. Each value represents the amount of protein relative to that of the control group transfected with the scrambled siRNA in the same set of experiments. Three to five independent experiments were run in each experiments and results were presented as means ± SD. *P < 0.05 *vs.* the Control group, ^#^ *P* < 0.05 *vs.* the High glucose group

Txnip has been reported to play central roles in diabetes progression particularly in relation to β-cell apoptosis (11). In the present study, *S-Equol* was proved to reduce INS-1 cell apoptosis. To verify the relation between *S-Equol* and Txnip, with western blotting analysis, we found that *S-Equol* significantly inhibited Txnip increase in ZDF rats (Fig. 4). Similar results were seen in Txnip protein and mRNA levels in *S-Equol* administrated high glucose cultured INS-1 cells (Fig. 5 a-c). In addition, we found that this *S-Equol* inhibition of the glucose-induced Txnip over expression occurred at the transcriptional levels as demonstrated by the decrease in the *Txnip* promoter-driven luciferase activity (Fig. 5d).

The transcription complex Chrebp/Mlx is determinant for the induction of *Txnip* genes (15, 20, 21, 26). As shown in Fig. 4a-b, Chrebp and Mlx protein in ZDF rats were partially inhibited in ZDF rats and in INS-1 cells with *Equol* treatment compared with those without *Equol*. Moreover, we found that *S-Equol* treatment decreased Chrebp occupancy of the *Txnip* promoter in vitro as demonstrated by ChIP assay, whereas the IgG control showed nearly no enrichment confirming the specificity of these ChIP assays (Fig. 5 e). Furthermore, transfection of INS-1 cells with *Chrebp* small interfering RNAs (Fig. 5 f-h) not only resulted in effective Chrebp knockdown, but also led to a dramatic reduction in Txnip expression, further confirming the important role Chrebp plays in *S-Equol* regulating Txnip transcription. Taken together, S-Equol suppresses Chrebp/Txnip signaling in vivo and in vitro.

### *S-Equol* regulates Chrebp/Txnip signaling via modulating PKA/PP2A activities

**Fig. 6:**
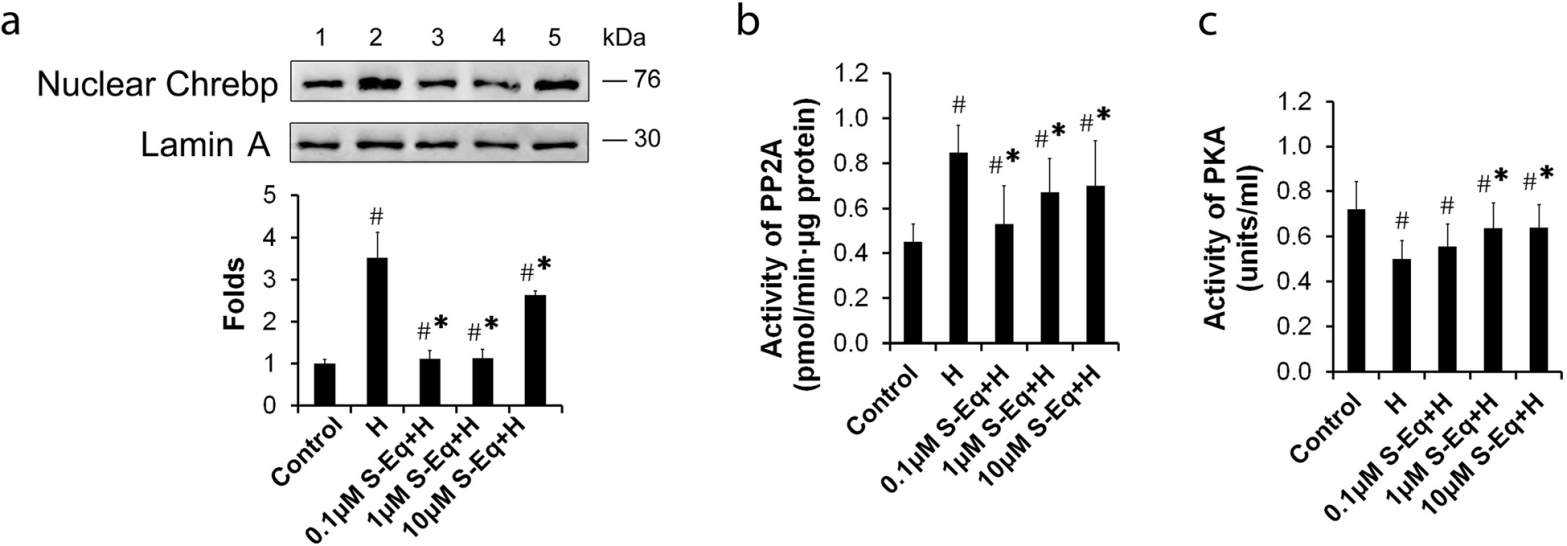
*S-Equol* modulates PKA/PP2A activities in high glucose cultured INS-1 cells. INS-1 cells were treated for 48 h with or without *S-Equol* (0.1, 1 and 10 μM) in the continuous presence of high glucose (26.2 mM), and cells cultured with 5 mM of glucose denote the control. (**a**) Western blotting analysis of nuclear Chrebp; (**b**) The PP2A and (**c**) PKA activities. H, High glucose; S-Eq*, S-Equol;* Lane “1 – 5” represents Control, H), 0.1 μM *S-Eq* + H, 1 μM *S-Eq* + H and 10 μM *S-Eq* + H group respectively; PP2A,protein phophatase 2A; PKA, protein kinase A. Three to five independent experiments were run in each experiments and results were presented as means ± SD. *P < 0.05 *vs.* the Control group, ^#^ *P* < 0.05 *vs.* the High glucose group

Nuclear translocation of Chrebp is one of the important processes in the glucose activation of *Txnip* gene transcription (15, 21, 28). To determine whether *S-Equol*-mediated inhibition in *Txnip* gene expression was correlated to a decrease in nuclear Chrebp content, Western blot analysis was performed using nuclear protein extracts from INS-1 cells cultured in high glucose with the presence or absence of *S-Equol* (Fig. 6a). Incubation of INS-1 cells with glucose led to a 3.4-fold increase in Chrebp content in the nucleus. In contrast, after *S-Equol* treatment, the Chrebp translocation to the nucleus was partially inhibited.

Translocation of Chrebp into the nucleus has been shown to require a number of dephosphorylation steps, in particular dephosphorylation of Ser^196^ by PP2A (19). We therefore investigated the activity of PP2A, and indeed found a clear decline in high-glucose cultured INS-1 cells with *S-Equol* treatment (Fig. 6b). Moreover, it was proposed that Chrebp/Mlx heterodimer forming and DNA binding resulted from dephosphorylation Ser^666^ (phosphorylated by PKA) (28), directly affecting the transcriptional efficacy. We therefore determined the activity of PKA and results showed that *S-Equol* significantly enhance the PKA activity in high-glucose cultured INS-1 cells (Fig. 6c). Overall, these results suggested that *S-Equol*-mediated inhibition of Chrebp/Txnip signaling was partially related to modulate the PKA and PP2A activities.

## Discussion

In the present study, we confirmed the anti-diabetic activity of S-*Equol* in ZDF rats, a type 2 diabetic animal model, and clarified the regulatory mechanism of insulin secretion by *S-Equol* in relation to influence the Chrebp/Txnip signaling *via* modulating the activities of PP2A and PKA in high glucose cultured INS-1 cells. The current results provide a novel mechanism for elucidating the anti-diabetic effect of *S-Equol*.

*S-Equol* is the main active product of daidzein metabolism, which is produced by specific microflora in the gut. “*Equol* hypothesis”, proposed by Setchell et al. (6), states that *Equol* producers (organisms possessing microbiota that can produce *S-Equol*) receive maximal health benefits from soy intake. *S-Equol* has a stronger estrogenic effect than daidzein, leading to the relief of menopausal symptoms and the prevention of prostate cancer, which supports the *Equol* hypothesis (4, 6, 29). Notably, recent evidences, including *S-Equol* supplementation decreased glycated hemoglobin concentrations in non-*S-Equol*-producing subjects (8, 9) and racemic *Equol* administration suppressed hyperglycemia in ob/ob mice (10), alloxan-induced mice and STZ-induced rats (3), all support that *S-Equol* was a powerful metabolic regulators even in *Equol* non-producers and the potential mechanism was beyond the estrogenic effect. In this study, *Equol* supplementation were also identified to substantially improve diabetic phenotypes in ZDF rats. Firstly, it is known that the weight loss and the increase of water intake that occurs unintentionally were warning signs of aggravating diabetes (30). ZDF rats treated with *Equol* still kept gaining weight whereas the weight loss occurred in ZDF rats at 14th week age. Meanwhile, *Equol* supplementation restricted the water intake of ZDF rats. Secondly, *Equol* significantly reduced the increase of glucose levels of ZDF rats in the whole treatment period. Admittedly, the concentrations of glucose of ZDF rats treated with *Equol* remained to be higher than normal. This might partially attributed to the feature of the ZDF rat, which has a missense mutation in the gene coding the leptin receptor (fa/fa) and spontaneously develops T2DM with severe diabetic phenotypes including higher glucose levels (31). Thirdly, *Equol* improved the lipid profile of ZDF rats. In line with our results, lower prevalence of dyslipidemia was observed in *Equol* producers (32) and reduced lipid levels were also found in *Equol* treated ob/ob mice (33), and further studies revealed the anti-hyperlipemia effect of *Equol* was correlated with the inhibition of hepatic lipid synthesis and the modulation of intestinal lipid absorption. As mounting evidences demonstrated *Equol* given regularly was capable to improve symptoms of diabetes and be a supportive therapy in the treatment of T2DM, further study to unravel the underlying mechanism were need.

Defects in insulin secretion are a critical determinant of the risk and progression of T2DM (1, 11). The total number of β-cells (related to proliferation and survival) and glucose response in individual β-cells are responsible for insulin secretion (1). In this study, we showed that *S-Equol* increased glucose-stimulated insulin secretion in vivo and in vitro, which might correlated with both β-cell proliferation increase and β-cell apoptosis inhibition. In line with our results, previous studies (2, 3, 10) also identified that S-*Equol* could prevent oxidative stress-induced cell death in INS-1 cells and enhance β-cell proliferation in STZ-induced diabetic rats. Besides inhibiting loss of β-cells, S-*Equol* was also found to play a role in enhancing insulin synthesis as evident by significantly elevated C-peptide in ZDF rats and increased cellular *PPI* mRNA in high-glucose cultured INS-1 cells. Interestingly, concerning the effects on *PPI* mRNA transcription in INS-1 cells, S-*Equol* at high dosage (10 μM) was not better than that at low dosage (0.1, 1 μM). Insulin synthesis was regulated at both the transcriptional and translational level (34), and *PPI* mRNA transcription was regulated by many trans-activators including paired box gene 6, pancreatic and duodenal homeobox-1, MafA, and β-2/Neurogenic differentiation 1. The underlying mechanism of the insulin synthesis modulation by *S-Equol* might be complicated and further investigations were needed to explain these phenomena.

Txnip is a protein with multifunctional roles in diverse cellular responses such as proliferation, apoptosis and differentiation (14). Recently, Txnip was proved to be essential for glucotoxicity-induced β-cell death, whereas lack of Txnip promotes endogenous β-cell survival and prevents type 1 and type 2 diabetes (11, 21). In this study, we found that the content of Txnip protein was significantly reduced by *S-Equol* in ZDF rats and high glucose cultured INS-1 cells. Consistent with our results, some other soy derived isoflavones including genistein (35) and Biochanin A (36), and resveratrol (37, 38) have also been proved to inhibit Txnip expression, however, there was no further investigation about the mechanisms. Generally, the cellular Txnip level is regulated at the transcriptional level, post-translational level and degradation rate (27). In the present study, *Txnip* mRNA levels dropped by over 50% and significantly decrease in the *Txnip* promoter transcription activity were determined in high-glucose cultured INS-1 cells with *S-Equol* treatment, which suggests that *S-Equol* mediated drop in Txnip levels is likely due to transcriptional repression of *Txnip*.

Glucose-induced Txnip expression is mediated via a nonpalindromic E-box motif consisting of a repeat of CACGAG sequences in the *Txnip* promoter that serves as the binding site for the Chrebp (21, 26, 28). The basic helix-loop-helix transcription factor Chrebp has been recognized as the main transcription factor mediating glucose-induced gene expression in liver as well as in β-cells, whereas its paralog, Mondo A, has been shown to do the same in muscle, and a recently discovered isoform, Chrebp-β, has been described in adipocytes (23). In the present study, as evidenced by a significant Txnip reduction concomitant with Chrebp knocking down by *si-chrebp*, the relationship between Chrebp and Txnip was confirmed. Furthermore, the decreased robust Chrebp/Mlx protein content and Chrebp occupancy on the *Txnip* promoter were observed in high-glucose cultured INS-1 cells with *S-Equol* treatment, which indicates that *S-Equol* could inhibit the transcription factor Chrebp binding to the E-box of the *Txnip* promoter.

Translocation of ChREBP from the cytosol to the nucleus in response to elevated glucose concentrations has been documented in both liver (39) and clonal pancreatic β-cells (21, 25, 40, 41) and is likely to be a key regulatory step in the activation of downstream genes (28). Our study demonstrates that *S-Equol* not only decreases Chrebp expression but also reduces the translocation of Chrebp from the cytosol to the nucleus. Previous studies have proved that glucose-dependent Chrebp nuclear translocation is subject to a series of phosphorylation/dephosphorylation reactions. In the liver, while in fasting and in low glucose conditions, Chrebp is phosphorylated by PKA on S^196^ and T^666^ and by AMP-activated protein kinase on binding; by contrast, in high glucose conditions, PP2A activation by xylulose 5-phosphate induces dephosphrylation of S^196^, T^666^, and S^568^, nuclear import and DNA binding (19). Moreover, recent studies also confirmed that, in the β-cell, elevated glucose concentrations are partially associated with PP2A inhibition and the elevation of the intracellular cAMP levels and PKA signaling (3, 28). In the present study, *S-Equol* was proved to activate PKA and inhibit PP2A in high-glucose cultured INS-1 cells, which might directly affect the Chrebp/Mlx heterodimers forming and DNA binding and thereby reduce the *Txnip* transcription. Consistent with that, Hiroko et al. (3) had reported that treatment with a PKA inhibitor diminished *S-Equol*–mediated effects on β-cell proliferation and glucose-stimulated insulin secretion in streptozotocin-induced hyperglycemia rats, and oxidative stress (alloxan)-induced cell death. These results further demonstrated that *S-Equol* ameliorates insulin secretion failure through Chrebp/Txnip signaling via modulating PKA/PP2A activities.

## Conclusion

To our knowledge, this is the first report to illustrate the relationship between Txnip and the anti-diabetic effects of *S-Equol*. Moreover, in the present study, the serum *S-Equol* concentration after 6 weeks of 120 mg/kg racemic Equol gavage in ZDF rats was about 7 mmol/L, and the effective dose observed in INS-1 cells was ranged from 0.1 to 10 μM. Given that blood *S-Equol* concentrations are > 1 mM in some *S-Equol* producers (6, 29), our results further confirmed that taking an *S-Equol* supplement has the potential to prevent T2DM and relieve the prediabetic symptoms.

## Data Availability

All data generated or analyzed during this study are included in this published article or are available from the corresponding author on reasonable request.

## Abbreviations

AUC: Areas under the curve
BIS: Basic insulin secretion
ChIP: Chromatin immunoprecipitation
Chrebp: Carbohydrate response element-binding protein
Eq: Equol
GSIS: Glucose-stimulated insulin secretion
HDL: High density lipoprotein
LDL: Low density lipoprotein
OGTT: Oral glucose tolerance test
PI: Propidium iodide
PKA: Protein kinase A
PP2A: Protein phophatase
PPI: Preproinsulin
QRT-PCR: Quantitative real-time PCR
T2DM: Type 2 diabetes mellitus
Tch: Total cholesterol
TG: Total triglyceride
TUNEL: Terminal deoxynucleotidyl transferase dUTP nick end labeling
Txnip: Thioredoxin-interacting protein
ZDF: Zucker diabetic fatty
ZLR: Zucker lean
